# Preoperative Aortic Calcification Volume Predicts Postoperative Complications in Nonpancreatic Cancer Patients Undergoing Pancreaticoduodenectomy

**DOI:** 10.1002/ags3.70132

**Published:** 2025-11-27

**Authors:** Masaki Horiuchi, Kentaro Miyake, Kota Sahara, Jun Yamamoto, Yutaro Kikuchi, Yasuhiro Yabushita, Yu Sawada, Yuki Homma, Ryusei Matsuyama, Itaru Endo

**Affiliations:** ^1^ Department of Gastroenterological Surgery Yokohama City University Graduate School of Medicine Yokohama Kanagawa Japan

**Keywords:** abdominal aortic calcification, pancreatic fistula, pancreaticoduodenectomy, postoperative complications

## Abstract

**Background/Objectives:**

Postoperative complications following pancreaticoduodenectomy (PD) remain high, particularly in patients with soft pancreatic texture. Abdominal aortic calcification volume (AACV), a surrogate marker of systemic arteriosclerosis, has been associated with increased surgical risk in lower gastrointestinal procedures; however, its relevance in hepatopancreatobiliary surgery has not been well established. This study aimed to evaluate the clinical significance of AACV in predicting postoperative complications after PD.

**Methods:**

We retrospectively analyzed 182 patients who underwent PD for nonpancreatic malignancies at our institution between January 2007 and December 2023. AACV was measured using the SYNAPSE VINCENT 3D imaging system, assessing calcification from the renal artery to the common iliac artery bifurcations on preoperative CT. Clinical characteristics and short‐term postoperative outcomes were evaluated.

**Results:**

The cohort included distal cholangiocarcinoma (57.1%), papillary carcinoma (35.7%), duodenal cancer (2.7%), IPMN (2.2%), and pancreatic neuroendocrine tumors (1.6%). Soft pancreatic texture was observed in 85.2% of patients. The median AACV was 894.5 mm^3^ (range: 284.6–2951.9) and correlated weakly with age (*r* = 0.377, *p* < 0.001). Patients with Clavien–Dindo grade≥IIIa complications had significantly higher AACV than those without (1745.9 vs. 751.9 mm^3^, *p* < 0.001). AACV was identified as an independent predictor of both severe complications (HR: 10.9; 95% CI: 4.44–26.3) and POPF≥Grade B (HR: 4.85; 95% CI: 2.2–10.8).

**Conclusion:**

AACV is significantly associated with severe postoperative complications and clinically relevant pancreatic fistula following PD, particularly in patients with nonpancreatic malignancies. It may serve as a valuable preoperative risk predictor in this population.

## Abstract 2

This study evaluated the association between abdominal aortic calcification volume (AACV) and postoperative complications following pancreaticoduodenectomy (PD) for non‐pancreatic malignancies. AACV was identified as an independent predictor of clinically relevant postoperative pancreatic fistula, and its addition to the Fistula Risk Score improved predictive performance. These findings support the potential of AACV as a valuable preoperative risk marker.

## 1 Introduction

Pancreaticoduodenectomy (PD) remains one of the most complex and invasive procedures in gastrointestinal surgery, with complication rates still reported to remain as high as 30%–40% [1]. Despite recent advancements in minimally invasive approaches such as laparoscopic and robotic techniques, the incidence of postoperative complications after PD has not significantly decreased [2]. Among these complications, postoperative pancreatic fistula (POPF) is one of the most critical, as it may lead to life‐threatening sequelae such as intraperitoneal bleeding caused by pseudoaneurysm rupture [3]. Therefore, identifying novel preoperative predictors of adverse outcomes following PD is of utmost importance.

A soft pancreatic texture, often associated with the absence of main pancreatic duct obstruction, has been identified as a major risk factor for POPF [4]. According to the report by John W. Lin et al., a soft pancreatic texture was associated with a fistula rate of 22.6%, and the risk of developing fistula was 20.4 times higher compared to patients with a firm pancreas (95% confidence interval, 4.7–90.9) [5]. Although various measures have been implemented, no method has yet proven to be definitively effective. Some researchers have proposed two‐stage pancreaticoduodenectomy as one of the few investigational strategies available [6]. However, there are still many unknowns regarding the risk factors for POPF in patients undergoing PD.

With an aged population, the number of elderly patients undergoing major abdominal surgeries, including PD, is increasing. These patients often present with systemic arteriosclerosis, which has been associated with poorer postoperative outcomes compared to younger patients. Several previous studies have demonstrated that PD is associated with an increased risk of postoperative mortality and complications in patients aged 60–65 years and older, and is further correlated with higher postoperative morbidity and prolonged length of hospital stay (LOS) in those aged 70 years and above [7, 8]. Given the inherently high‐risk nature of PD, careful patient selection is essential.

Abdominal aortic calcification (AAC), a radiographic marker of systemic atherosclerosis, has been reported to be associated with a history of cardiovascular diseases with adverse outcomes, including coronary artery disease and abdominal aortic aneurysm, as well as with a history of smoking and pathological fractures [9, 10]. Furthermore, recent studies have suggested that the volume of AAC (AACV) is also associated with surgical outcomes [11]. However, the significance of preoperative AAC measurement in the hepatobiliary‐pancreatic surgery remains unclear.

In this study, we aimed to investigate the relationship between preoperative AAC and short‐term postoperative outcomes in patients undergoing PD.

## 2 Materials and Methods

### 2.1 Patients’ selection

We retrospectively analyzed 182 patients who underwent PD at the Yokohama City University (YCU) Hospital between 2007 and 2023. These cases included 104 cases of distal bile duct cancer, 65 cases of ampullary carcinoma, five cases of duodenal cancer, and four cases of intraductal papillary mucinous neoplasm (IPMN). Pancreatic cancer cases with a low risk of POPF were excluded from this study due to the predominance of hard pancreatic texture. Additionally, cases that underwent simultaneous liver resection were also excluded from this study.

Preoperative factors included age, sex, body mass index (BMI), pathological diagnosis, and a history of atherosclerosis‐related comorbidities, such as hypertension, coronary artery disease, cerebrovascular disease, and diabetes mellitus. Intraoperative assessments comprised evaluation of the pancreatic parenchymal texture (categorized as soft or hard) and measurement of the main pancreatic duct (MPD) dilatation, defined as a duct diameter exceeding 3 mm. In addition, as an evaluation of obesity contributing to arteriosclerosis, the subcutaneous fat area and visceral fat area at the umbilical level were also measured from preoperative CT images. The perioperative factors included operative time, estimated blood loss, the necessity for blood transfusion, and anastomotic type. The study protocol was approved by the Institutional Ethics Committee (Yokohama City University; approval no. F250100031).

### 2.2 Evaluation of AAC by CT images

Abdominal CT scans were performed using 64‐slice multidetector CT systems as part of the standardized preoperative assessment. Abdominal aortic calcification (AAC) was quantified using SYNAPSE VINCENT software (FUJIFILM, Tokyo, Japan). The AAC volume (AACV) was defined as the total volume of calcification within the abdominal aorta, measured from the level of the renal artery bifurcation to the bifurcation of the common iliac arteries, in accordance with previous AAC evaluations [12]. The threshold for calcified lesions was set to a CT density of 130 Hounsfield Units (HU) or higher. The AACV was manually selected in the 3D image, and the volume of the calcified lesions was automatically calculated by SYNAPSE VINCENT software.

### 2.3 Statistical analysis

All statistical analyses were performed using EZR version 1.61 (Saitama Medical Center, Jichi Medical University, Saitama, Japan), which is a graphical user interface for R (The R Foundation for Statistical Computing, Vienna, Austria) [13]. Statistical significance of differences was determined by the Mann–Whitney U test or Pearson's correlation coefficient, as appropriate. The receiver operating characteristic (ROC) curve was used to determine the cut‐off value for each continuous variable.

To evaluate the incremental predictive value of AACV when added to the Fistula Risk Score (FRS), we constructed two logistic regression models: one with FRS alone and another with both FRS and AACV as predictors. Predictive performance was assessed using ten‐fold cross‐validation, and the area under the ROC curve (AUC) was calculated for each fold. The statistical significance of the difference in AUCs between models was evaluated using a paired *t*‐test. Statistical significance was set at *p* < 0.05.

## 3 Results

### 3.1 Patients’ characteristics

The characteristics of the 182 patients were summarized in Table 1. The average age was 70.3 years, and there were 135 males (74.2%). There were 77 patients (43.0%) with HT, 48 patients (26.8%) with HL and DM, and 114 patients (63.4%) with cardiovascular disease, with a median Brinkman Index of 380. The average PNI was 46.5, median BMI was 22.2, and median VFA/SFA was 75.9 cm^2^/99.7 cm^2^. Soft pancreatic parenchyma was observed in 155 cases (85.2%), and the median AACV was 1292.7 mm^3^. The median operative time was 539 min, and the median blood loss was 781 mL. Pancreatojejunostomy was performed in 157 cases (86.7%), while 25 cases (13.3%) involved pancreatogastrostomy. Postoperative complications occurred in 121 cases (66.9%), with 74 cases (40.7%) classified as Clavien‐Dindo grade≥IIIa, and 38cases (20.9%) presenting with POPF≥Grade B. The median hospital stay was 28 days.

**TABLE 1:** Patient demographics and clinical characteristics stratified by abdominal aortic calcification volume (AACV) (*n* = 182).

	Group A (AACV ≥ 2418.53 mm^3^) *n* = 58	Group B (AACV < 2418.53 mm^3^) *n* = 124	*p*
±10.2	72.2 ± 8.3	69.4 ± 10.9	0.189
74.2%	48 (82.8%)	87 (70.2%)	0.101
19.9–24.2	22.7 (20.9–24.6)	21.8 (19.5–23.8)	0.043
±0.5	3.8 ± 0.4	3.9 ± 0.5	0.435
±6.1	22.1 ± 5.4	22.5 ± 6.3	0.814
18.4–29.0	22.8 (18.7–27.3)	22.9 (18.4–29.2)	0.813
9.1–9.5	9.4 (9.2–9.5)	9.2 (9.0–9.5)	0.089
82–169	144 (95–192)	101 (72–150)	0.008
±49.2	185.2 ± 50.9	198.2 ± 47.8	0.078
0.50–1.19	0.72 (0.50–1.12)	0.73 (0.50–1.19)	0.541
5.4–6.3	5.7 (5.4–6.3)	5.8 (5.4–6.3)	0.596
±5.9	46.2 ± 6.2	46.4 ± 5.7	0.994
0–800	480 (0–800)	300 (0–775)	0.348
43.0%	27 (46.6%)	50 (40.3%)	0.424
26.8%	22 (37.9%)	26 (21.0%)	0.019
26.8%	15 (25.9%)	33 (26.6%)	1.000
63.4%	41 (70.7%)	73 (58.9%)	0.135
284.6–2951.9	4788.7 (3259.3–5727.7)	491.9 (182.0–1354.3)	< 0.001
33.0–121.4	97.4 (29.2–137.9)	75.0 (34.5–126.4)	0.585
58.6–131.8	87.4 (42.5–98.8)	80.4 (50.5–115.0)	0.978
448–632	564 (456–660)	533 (442–625)	0.578
475–1273	873 (548–1326)	707 (430–1229)	0.140
22.8%	13 (22.4%)	28 (22.6%)	1.000
13.3%	5 (8.6%)	20 (16.1%)	0.104
86.7%	53 (91.4%)	104 (83.9%)	0.104
64.3%	34 (58.6%)	83 (66.9%)	0.320
85.2%	52 (89.7%)	103 (83.1%)	0.273
66.9%	49 (84.5%)	72 (58.1%)	< 0.001
40.7%	39 (67.2%)	35 (28.2%)	< 0.001
20.9%	31 (53.5%)	7 (5.7%)	< 0.001
19.2%	13 (22.4%)	22 (17.7%)	0.545
13.7%	7 (12.1%)	18 (14.5%)	0.818
3.3%	3 (5.2%)	3 (2.4%)	0.593
6.0%	5 (8.6%)	6 (4.8%)	0.331
5.5%	2 (3.5%)	8 (6.5%)	0.506
1.7%	0 (0.0%)	3 (2.4%)	0.552
2.8%	1 (1.7%)	4 (3.2%)	1.000
3.9%	1 (1.7%)	6 (4.8%)	0.433
1.7%	0 (0.0%)	3 (2.4%)	0.552
19–41	34 (22–46)	25 (18–38)	0.010

To further examine the clinical significance of AACV, patients were stratified into two groups using a cutoff value of 2418.53 mm^3^, which corresponded to the optimal threshold derived from ROC analysis. As shown in the Table 1, patients in the high‐AACV group ≥ 2418.53 mm^3^ were older and had higher BMI compared to those in the low‐AACV group (< 2418.53 mm^3^). The high‐AACV group also showed a significantly greater incidence of postoperative complications, including POPF≥Grade B (*p* < 0.001), and a longer median hospital stay (*p* = 0.010).

### 3.2 Relationship between arteriosclerosis‐related parameters and AACV

**FIGURE 1:**
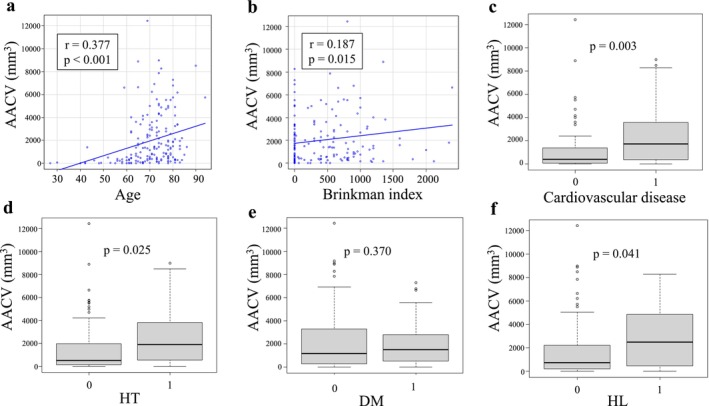
Association between arteriosclerosis‐related parameters and AACV. a: AACV showed a weak positive correlation with age (*r* = 0.377, *p* < 0.001). b: AACV was also weakly correlated with the Brinkman index (*r* = 0.187, *p* = 0.015). c: AACV was significantly elevated in patients with a history of cardiovascular disease (*p* = 0.003). d: Patients with a history of HT had significantly higher AACV than those without (*p* = 0.025). e: There was no significant difference in AACV between patients with and without DM (*p* = 0.370). f: AACV was significantly higher in patients with HL compared to those without (*p* = 0.041).

The AACV tended to increase with age, with a weak correlation coefficient of 0.377 (*p* < 0.001) (Figure 1a). There was no significant correlation with the preoperative Brinkman index, D‐dimer, triglycerides, and total cholesterol. The AACV was predominantly high in males (1441.1 mm^3^ vs. 478.5 mm^3^, *p* = 0.004) and predominantly elevated in HT (1714.7mm^3^ vs. 772.5mm^3^, *p* = 0.025), HL (1930.7 mm^3^ vs. 882.1 mm^3^, *p* = 0.041), and history of cardiovascular disease (1562.2 mm^3^ vs. 532.2 mm^3^, *p* = 0.003) (Figure 1b–e).

### 3.3 Association of AACV with postoperative complications

**FIGURE 2:**
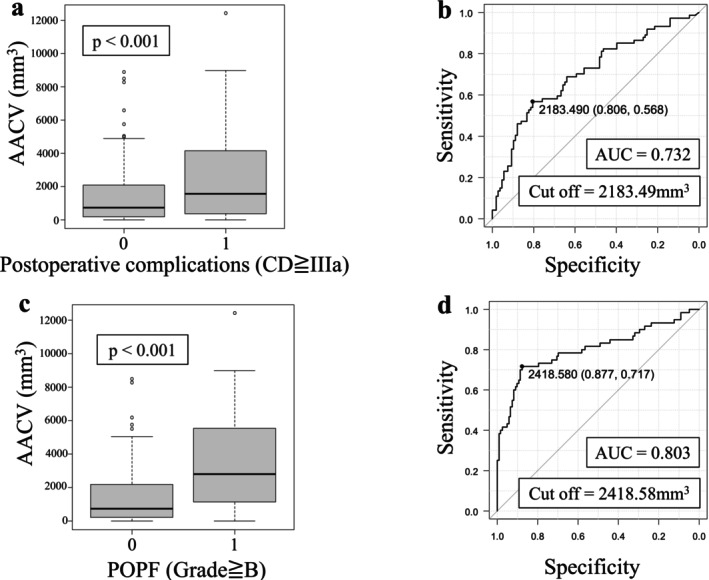
Association of AACV with postoperative complications. a: The AACV was significantly correlated with all postoperative complications (Clavien‐Dindo≥IIIa). b: The optimal cut‐off value of AACV to diagnose postoperative complications (Clavien‐Dindo≥IIIa), as determined by the ROC curve, was 2183.5 mm^3^ (area under the curve [AUC], 0.732). c: The AACV was significantly correlated with all postoperative pancreatic fistula (≥ Grade B). d: The cut‐off value of the AACV to diagnose postoperative pancreatic fistula (≥ Grade B), as determined by the ROC curve, was 2418.6 mm^3^ (area under the curve [AUC], 0.803).

The AACV in the Clavien‐Dindo grade≥IIIa group was significantly higher than that in the Clavien‐Dindo grade < IIIa group (2526.5 mm^3^ vs. 758.4 mm^3^, *p* < 0.001) (Figure 2a). The cutoff value of AACV for predicting postoperative complications classified as Clavien–Dindo grade IIIa or higher, as determined by receiver operating characteristic (ROC) curve analysis, was 2183.49 mm^3^ (area under the curve [AUC] = 0.732) (Figure **2** **b**). The median AACV in the POPF≥Grade B group was 3496.2 mm^3^, and the POPF<Grade B group was 698.3 mm^3^. AACV was significantly correlated with POPF≥Grade B (*p* < 0.001) (Figure 2c). The ROC curve analysis established the cutoff value of AACV for diagnosing POPF≥Grade B at 2418.58 mm^3^ (AUC = 0.802) (Figure 2d). To identify risk factors for postoperative complications (Clavien‐Dindo≥IIIa), univariate and multivariate logistic regression analyses were conducted (Table 2). Preoperative PNI, VFA (cm^3^), and AACV (mm^3^) showed significant differences during univariate analysis. In multivariate analysis, VFA (RR 2.160; 95% CI 1.11–4.22; *p* = 0.024) and AACV (RR 4.840; 95% CI 2.41–9.70; *p* < 0.001) were independent predictors. For predicting risk factors associated with POPF≥Grade B, univariate and multivariate logistic regression analyses were performed (Table 3). Significant differences were noted in preoperative VFA, soft pancreatic parenchyma, pancreatogastrostomy, and AACV during univariate analysis. VFA (RR 2.540; 95% CI 1.00–6.42; *p* = 0.049), soft pancreatic parenchyma (RR 5.050; 95% CI 1.11–22.9; *p* = 0.036), pancreatogastrostomy (RR 0.077; 95% CI 0.01–0.79; *p* = 0.031), and AACV (RR 15.700; 95% CI 6.34–38.7; *p* < 0.001) were independent predictors in multivariate analysis.

**TABLE 2:** Univariate and multivariate analysis of factors of postoperative complications (Clavien‐Dindo≥IIIa).

	Univariate analysis			Multivariate analysis		
	RR	95% CI	*p*	RR	95% CI	*p*
Age	1.31	0.72–2.39	0.373			
Sex (Male/Female)	1.66	0.82–3.34	0.159			
BMI	1.60	0.76–3.38	0.221			
PNI	1.83	1.00–3.34	0.049	2.16	1.11–4.22	0.024
VFA (cm^2^)	1.87	1.02–3.44	0.044	1.79	0.91–3.52	0.093
SFA (cm^2^)	1.00	0.99–1.01	0.216			
Soft pancreatic parenchyma	1.23	0.51–2.96	0.643			
AACV (mm^3^)	3.32	1.73–6.39	< 0.001	4.84	2.41–9.70	< 0.001
Operative time (min)	1.73	0.95–3.15	0.071			
Blood loss (mL)	1.58	0.87–2.87	0.132			
Pancreatogastrostomy	0.69	0.28–1.71	0.421			

Abbreviations: AACV, abdominal aortic calcification volume; BM, body mass index; PNI, prognostic nutritional index; SFA, subcutaneous fat area; VFA, visceral fat area.

**TABLE 3:** Univariate and multivariate analysis of factors of POPF (Grade *≥* B).

	Univariate analysis			Multivariate analysis		
	RR	95% CI	*p*	RR	95% CI	*p*
Age	1.19	0.64–2.22	0.589			
Sex (male)	2.18	0.99–4.75	0.051			
BMI	2.1	0.98–4.49	0.056			
PNI	0.78	0.42–1.45	0.428			
VFA (cm^2^)	2.27	1.21–4.29	0.011	2.54	1.00–6.42	0.049
SFA (cm^2^)	1.65	0.88–3.08	0.116			
Soft pancreatic parenchyma	4.76	1.36–16.7	0.015	5.05	1.11–22.9	0.036
AACV (mm^3^)	18	8.28–39.3	< 0.001	15.7	6.34–38.7	< 0.001
Operative time (min)	1.49	0.80–2.78	0.208			
Blood loss (mL)	1.65	0.88–3.08	0.116			
Pancreatogastrostomy	0.07	0.01–0.56	0.012	0.08	0.01–0.79	0.031

### 3.4 Predictive performance of FRS alone versus combined FRS + AACV model

**FIGURE 3:**
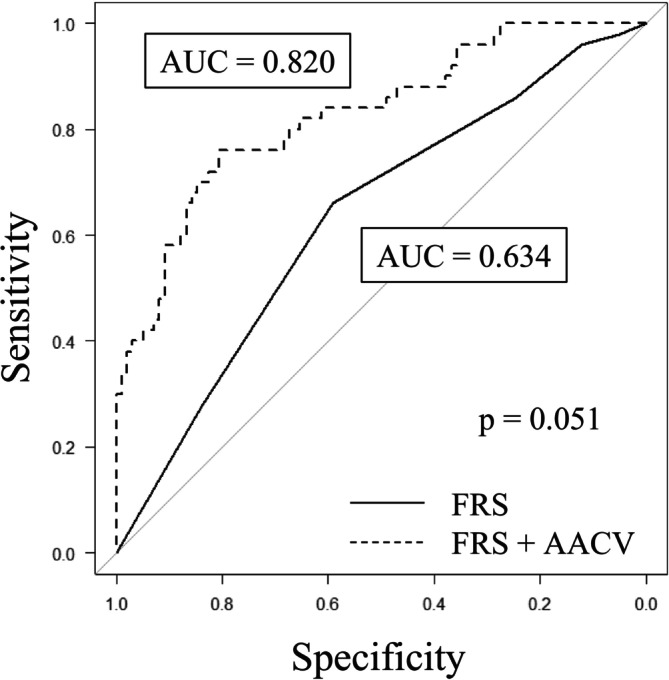
ROC curves comparing predictive performance of FRS‐only and FRS + AACV models. The model using FRS alone showed modest discriminatory ability (AUC = 0.634), while the model combining FRS and AACV demonstrated improved performance (AUC = 0.820). The difference in AUCs showed a trend toward statistical significance (*p* = 0.051, paired *t*‐test).

The model using FRS alone demonstrated a mean AUC of 0.634, whereas the model incorporating both FRS and AACV achieved a higher mean AUC of 0.820. The difference in AUCs approached statistical significance (*p* = 0.051) (Figure 3).

## 4 Discussion

This study demonstrated that AACV is significantly associated with both overall postoperative complications and the development of POPF in patients undergoing PD for nonpancreatic malignancies.

AACV is widely recognized as a surrogate marker of systemic atherosclerosis and has been associated with adverse cardiovascular outcomes, including coronary artery disease and abdominal aortic aneurysm [14, 15]. Large cohort studies have shown that abdominal aortic calcification correlated with increased all‐cause and cardiovascular mortality [9]. Additionally, systematic reviews have underscored the prognostic value of AAC in cardiovascular diseases [16].

Beyond cardiovascular risks, recent studies suggest that AACV is also linked to surgical outcomes. In cardiac surgery, AAC has been associated with perioperative complications and in liver transplantation, Imaoka et al. reported a relationship between high AACV and both increased complications and decreased survival [17, 18]. In hepatobiliary and pancreatic surgeries, AAC has been identified as a risk factor for complications, including POPF, particularly in PD performed for biliary tract or pancreatic malignancies [19, 20].

In gastrointestinal surgery, AAC has emerged as a potential predictor of anastomotic leakage [21, 22]. Preoperative CT‐based aortic calcification indices have shown significant correlation with both incidence and severity of leakage after anterior resection for rectal cancer [12]. These findings support the hypothesis that systemic atherosclerosis impairs tissue perfusion at surgical sites, contributing to healing disorders such as POPF or anastomotic leaks [23]. Mechanistically, aortic calcification reduces vascular compliance, leading to hemodynamic instability and reduced organ perfusion [24]. In the context of PD, such effects may result in ischemia at the pancreatic stump or anastomotic site, especially in patients with soft pancreatic tissue, thereby promoting POPF.

While previous studies, such as those by Watanabe et al. and Kakizawa et al., have highlighted the predictive value of AAC or AACV in hepatobiliary or pancreatic surgery, our study uniquely focuses on a homogeneous population of nonpancreatic cancer PD patients, in whom soft pancreatic texture is more prevalent and risk stratification is more challenging [19, 20]. Furthermore, we employed precise volumetric quantification of AACV using 3D imaging software, potentially improving reproducibility and facilitating integration into preoperative planning compared to prior calcification indices.

Although AACV may mechanistically contribute to impaired tissue perfusion at the anastomotic site, thereby promoting POPF, its association with nonanastomotic complications such as pneumonia or cholangitis is less directly explained. These findings may reflect underlying systemic frailty, including reduced cardiopulmonary reserve, chronic inflammation, or immunosenescence, which are not directly measured in this study. Therefore, AACV may be functioning as a surrogate marker for overall physiological vulnerability in elderly patients with comorbid conditions. Further studies incorporating frailty indices or cardiopulmonary functional tests are warranted to validate this hypothesis.

Given the clinical importance of POPF, identifying preoperative risk factors is critical. While well‐known predictors include a soft pancreatic texture and a narrow main pancreatic duct, our study emphasizes the role of AACV as a potential imaging biomarker [23, 25]. Notably, we excluded pancreatic cancer cases to focus on patients with softer pancreatic parenchyma, enhancing the clarity of our findings. AACV remained an independent predictor of both overall postoperative complications and clinically relevant POPF.

In addition, while our results highlight the prognostic significance of AACV, the clinical utility of this parameter will depend on the establishment of standardized cut‐off values. Previous studies, such as Shen et al., have used median values or ROC‐derived thresholds; however, such values may vary based on imaging modality, population demographics, and calcification distribution [12]. Future prospective studies should aim to define reproducible cut‐off points with high predictive accuracy to facilitate the use of AACV in clinical decision‐making.

Although the Fistula Risk Score (FRS) has been widely validated as an intraoperative risk model for POPF, its performance may be suboptimal in patients undergoing PD for nonpancreatic malignancies, where soft pancreatic texture is more prevalent [26]. In our cohort, the predictive ability of FRS alone was modest (mean AUC = 0.634).

In contrast, when AACV was incorporated into the predictive model alongside FRS, the mean AUC increased substantially to 0.820. While the difference in AUCs did not reach conventional levels of statistical significance (*p* = 0.051), it demonstrated a clear trend toward improved discrimination.

This finding suggests that AACV provides complementary risk information that is not captured by intraoperative variables alone. Because AACV reflects systemic vascular health, it may serve as a preoperative surrogate for impaired perfusion and tissue fragility, thereby augmenting the anatomical and technical factors already considered in FRS.

Integrating AACV into risk models could therefore enhance perioperative risk stratification, especially in nonpancreatic malignancy cases, and help tailor postoperative management strategies more precisely. The debate between pancreatogastrostomy and pancreatojejunostomy has persisted for a long time. Several meta‐analyses have reported a lower incidence of POPF with pancreatogastrostomy [27, 28]. However, other studies have shown no significant difference in POPF rates between the two reconstruction methods [29, 30]. This discrepancy in findings may be attributable to differences in patient backgrounds. As a secondary finding of the present study, it may be reasonable to actively consider pancreatogastrostomy in patients with a soft pancreas and high AACV, where its inhibitory effect on pancreatic fistula formation has been demonstrated.

From a clinical management perspective, patients with high AACV might benefit from more intensive postoperative care. This may include prolonged drain placement, use of somatostatin analogues, or closer biochemical and radiological monitoring. Stratification based on AACV could assist in optimizing perioperative decision‐making and resource utilization. Another practical advantage of AACV is that it can be derived from routine preoperative CT scans, which are already part of the standard surgical workup. This makes AACV a cost‐effective and accessible biomarker, particularly attractive for centers without access to advanced imaging or intraoperative risk assessment tools.

However, our study has several limitations. First, it is a retrospective analysis conducted at a single center, limiting external generalizability. Second, the sample size was modest, precluding more detailed subgroup analyses. Third, although AACV was measured using a standardized software system, consensus has not yet been reached regarding clinically relevant cut‐off values. Fourth, although our data suggest that high AACV is associated with increased risk of complications, this was an observational study, and we did not assess whether intensified postoperative interventions—such as prolonged drainage or administration of somatostatin analogs—actually reduce complication rates in high‐risk patients. Therefore, these strategies should be viewed as hypotheses for future prospective interventional studies, rather than immediate clinical recommendations.

## 5 Conclusion

In conclusion, AACV appears to be a promising preoperative risk factor for complications after PD, particularly for the development of POPF. These findings are especially relevant in patients undergoing PD for nonpancreatic malignancies, where soft pancreatic texture is more prevalent and surgical risk is inherently higher. Future prospective, multicenter studies with larger cohorts and standardized AACV measurement protocols are warranted to validate these findings and potentially integrate AACV into perioperative risk assessment strategies.

## Author Contributions

**Masaki Horiuchi:** conceptualization, writing – original draft, data curation, formal analysis. **Kentaro Miyake:** conceptualization, writing – original draft. **Kota Sahara:** data curation. **Jun Yamamoto:** formal analysis. **Yutaro Kikuchi:** writing – review and editing. **Yasuhiro Yabushita:** writing – review and editing. **Yu Sawada:** writing – review and editing. **Yuki Homma:** writing – review and editing. **Ryusei Matsuyama:** writing – review and editing. **Itaru Endo:** conceptualization, writing – original draft.

## Funding

This research did not receive any specific grant from funding agencies in the public, commercial, or nonprofit sectors.

## Ethics Statement

The study protocol was approved by the Ethical Advisory Committee of Yokohama City University Graduate School of Medicine (approval number: B200700094) and the Institutional Review Board of the participating hospital. An opt‐out method was used after disclosure of the study information instead of written informed consent.

## Conflicts of Interest

Authors declare no Conflicts of Interest for this article. Itaru Endo is an editorial board member of Annals of Gastroenterological Surgery.
