# Correction to “Preoperative Aortic Calcification Volume Predicts Postoperative Complications in Nonpancreatic Cancer Patients Undergoing Pancreaticoduodenectomy”

**DOI:** 10.1002/ags3.70262

**Published:** 2026-08-10

M. Horiuchi , K. Miyake , K. Sahara , et al., “Preoperative Aortic Calcification Volume Predicts Postoperative Complications in Nonpancreatic Cancer Patients Undergoing Pancreaticoduodenectomy,” Annals of Gastroenterological Surgery 10, no. 3 (2026): 803–810. 10.1002/ags3.70132 PMC1317828342146811

In the published version of this article, several rows in Table 1 were inadvertently omitted.

The correct version of Table 1 is provided below:

**TABLE 1:** Patients' characteristics.

	*n* = 182	Group A (AACV ≥ 2418.53 mm^3^) *n* = 58	Group B (AACV < 2418.53 mm^3^) *n* = 124	*p*
Age	70.3 ± 10.2	72.2 ± 8.3	69.4 ± 10.9	0.189
Sex, male	135 (74.2%)	48 (82.8%)	87 (70.2%)	0.101
Body mass index (kg/m^2^)	22.2 (19.9–24.2)	22.7 (20.9–24.6)	21.8 (19.5–23.8)	0.043
Albumin (g/dL)	3.8 ± 0.5	3.8 ± 0.4	3.9 ± 0.5	0.435
Prealbumin (mg/dL)	22.4 ± 6.1	22.1 ± 5.4	22.5 ± 6.3	0.814
Platelet count (*10^4^ μL)	22.9 (18.4–29.0)	22.8 (18.7–27.3)	22.9 (18.4–29.2)	0.813
Calcium (mg/dL)	9.3 (9.1–9.5)	9.4 (9.2–9.5)	9.2 (9.0–9.5)	0.089
Triglycerides (mg/dL)	109.5 (82–169)	144 (95–192)	101 (72–150)	0.008
Total cholesterol (mg/dL)	194.1 ± 49.2	185.2 ± 50.9	198.2 ± 47.8	0.078
D–dimer (μg/mL)	0.73 (0.50–1.19)	0.72 (0.50–1.12)	0.73 (0.50–1.19)	0.541
HbA1c (%)	5.7 (5.4–6.3)	5.7 (5.4–6.3)	5.8 (5.4–6.3)	0.596
PNI	46.3 ± 5.9	46.2 ± 6.2	46.4 ± 5.7	0.994
Brinkman Index	380 (0–800)	480 (0–800)	300 (0–775)	0.348
Hypertension	77 (43.0%)	27 (46.6%)	50 (40.3%)	0.424
Hyperlipidemia	48 (26.8%)	22 (37.9%)	26 (21.0%)	0.019
Diabetes mellitus	48 (26.8%)	15 (25.9%)	33 (26.6%)	1.000
Cardiovascular disease	114 (63.4%)	41 (70.7%)	73 (58.9%)	0.135
AACV (mm^3^)	1292.7 (284.6–2951.9)	4788.7 (3259.3–5727.7)	491.9 (182.0–1354.3)	< 0.001
Visceral fat area (cm^2^)	75.9 (33.0–121.4)	97.4 (29.2–137.9)	75.0 (34.5–126.4)	0.585
Subcutaneous fat area (cm^2^)	99.7 (58.6–131.8)	87.4 (42.5–98.8)	80.4 (50.5–115.0)	0.978
Perioperative findings				
Operative time(min)	539 (448–632)	564 (456–660)	533 (442–625)	0.578
Blood loss(mL)	781 (475–1273)	873 (548–1326)	707 (430–1229)	0.140
Blood transfusion	41 (22.8%)	13 (22.4%)	28 (22.6%)	1.000
Pancreatogastrostomy	25 (13.3%)	5 (8.6%)	20 (16.1%)	0.104
Pancreatojejunostomy	157 (86.7%)	53 (91.4%)	104 (83.9%)	0.104
Main pancreatic duct dilatation (> 3mm)	117 (64.3%)	34 (58.6%)	83 (66.9%)	0.320
Soft pancreatic parenchyma	155 (85.2%)	52 (89.7%)	103 (83.1%)	0.273
Postoperative complications	121 (66.9%)	49 (84.5%)	72 (58.1%)	< 0.001
Postoperative complications (Clavien–Dindo ≥ IIa)	74 (40.7%)	39 (67.2%)	35 (28.2%)	< 0.001
Pancratic fistula (Grade B)	38 (20.9%)	31 (53.5%)	7 (5.7%)	< 0.001
Abdominal abscess	35 (19.2%)	13 (22.4%)	22 (17.7%)	0.545
Delayed gastric emptying	25 (13.7%)	7 (12.1%)	18 (14.5%)	0.818
Biliary leakage	6 (3.3%)	3 (5.2%)	3 (2.4%)	0.593
SSI	11 (6.0%)	5 (8.6%)	6 (4.8%)	0.331
Venous thrombosis	10 (5.5%)	2 (3.5%)	8 (6.5%)	0.506
Cholangitis	3 (1.7%)	0 (0.0%)	3 (2.4%)	0.552
Pneumonia	5 (2.8%)	1 (1.7%)	4 (3.2%)	1.000
Intra‐abdominal hemorrhage	7 (3.9%)	1 (1.7%)	6 (4.8%)	0.433
Mortality	3 (1.7%)	0 (0.0%)	3 (2.4%)	0.552
Postoperative hospital stay (day)	28 (19–41)	34 (22–46)	25 (18–38)	0.010

This correction does not affect the results, interpretation, or conclusions of the article.

We apologize for this error.

